# Supplementary material for: A New Assessment of Robust Capuchin Monkey (Sapajus) Evolutionary History Using Genome-Wide SNP Marker Data and a Bayesian Approach to Species Delimitation
Source: Genes (Basel). 2023 Apr 25;14(5):970. doi: 10.3390/genes14050970 (PMC10218464; doi:10.3390/genes14050970)
Supplement: Supplementary file 1 [file genes-14-00970-s001.zip › TableS1.pdf]

Table S1 - Morphology-based and phylogenetic taxonomies of robust capuchin monkeys.

Elliot (1913) [8]	Hershkovitz (1949)[1] [9]	Cabrera (1957) [10]	Hill (1960) [1]	Groves (2001, 2005) [11,12]	Silva-Júnior (2001) [3]	Rylands et al. (2013) [7]	Lima et al. 2017[2] [5]	Lima et al. 2018[3] [13]
Cebus apella<br><br>***Cebus fatuellus***<br>*C. f. fatuellus*<br>*C. f. peruanus*<br><br>***Cebus macrocephalus***<br><br>***Cebus libidinosus***<br><br>***Cebus azarae***<br>*C. a. azarae*<br>*C. a. pallidus*<br><br>***Cebus frontatus***<br><br>***Cebus variegatus***<br><br>***Cebus versuta***<br><br>***Cebus cirrifer***<br><br>***Cebus crassiceps***<br><br>***Cebus caliginosus***<br><br>***Cebus vellerosus***	***Cebus apella***	***Cebus apella***<br>*C. a. apella*<br>*C. a. margaritae*<br>*C. a. macrocephalus*<br>*C. a. libidinosus*<br>*C. a. paraguayanus*<br>*C. a. pallidus*<br>*C. a. xanthosternos*<br>*C. a. versutus*<br>*C. a. nigritus*<br>*C. a. vellerosus*<br>*C. a. robustus*	***Cebus apella***<br>*C. a. apella*<br>*C. a. margaritae*<br>*C. a. fatuellus*<br>*C. a. peruanus*<br>*C. a. tocantinus*<br>*C. a. macrocephalus*<br>*C. a. libidinosus*<br>*C. a. cay*<br>*C. a. pallidus*<br>*C. a. frontatus*<br>*C. a. xanthosternos*<br>*C. a. nigritus*<br>*C. a. robustus*<br>*C. a. magnus*<br>*C. a. juruanus*<br>*C. a. maranonis*	***Cebus apella***<br>*C. a. apella*<br>*C. a. fatuellus*<br>*C. a. margaritae*<br>*C. a. macrocephalus*<br>*C. a. peruanus*<br>*C. a. tocantinus*<br><br>***Cebus libidinosus***<br>*C. l. libidinosus*<br>*C. l. pallidus*<br>*C. l. paraguayanus*<br>*C. l. juruanus*<br><br>***Cebus nigritus***<br>*C. n. nigritus*<br>*C. n. robustus*<br>*C. n. cucullatus*<br><br>***Cebus xanthosternos***	***Cebus (Sapajus) apella***<br><br>***Cebus (Sapajus) macrocephalus***<br><br>***Cebus (Sapajus) libidinosus***<br><br>***Cebus (Sapajus) cay***<br><br>***Cebus (Sapajus) nigritus***<br><br>***Cebus (Sapajus) robustus***<br>***Cebus (Sapajus) xanthosternos***	***Sapajus apella***<br><br>***Sapajus macrocephalus***<br><br>***Sapajus cay***<br><br>***Sapajus libidinosus***<br><br>***Sapajus nigritus***<br>*S. n. nigritus*<br>*S. n. cucullatus*<br><br>***Sapajus robustus***<br><br>***Sapajus xanthosternos***<br><br>***Sapajus flavius***	***Sapajus apella***<br><br>***Sapajus xanthosternos***<br><br>***Sapajus robustus***<br><br>***Sapajus nigritus***	***Sapajus apella***<br><br>***Sapajus libidinosus***<br><br>***Sapajus flavius***<br><br>***Sapajus xanthosternos***<br><br>***Sapajus robustus***<br><br>***Sapajus nigritus***

[1]Referent to the model hypothesis H0 in the Bayes factor species delimitation approach (Figure 3).

[2]Referent to the model hypothesis H1 in the Bayes factor species delimitation approach (Figure 3).

[3]Referent to the model hypothesis H2 in the Bayes factor species delimitation approach (Figure 3).
