# Supplementary material for: A New Assessment of Robust Capuchin Monkey (Sapajus) Evolutionary History Using Genome-Wide SNP Marker Data and a Bayesian Approach to Species Delimitation
Source: Genes (Basel). 2023 Apr 25;14(5):970. doi: 10.3390/genes14050970 (PMC10218464; doi:10.3390/genes14050970)
Supplement: Supplementary file 1 [file genes-14-00970-s001.zip › TableS2_NEW.pdf]

Table S2 - Samples used in the study. UT Austin: University of Texas at Austin; CPB/ICMBio: Centro Nacional de Pesquisa e Conservação de Primatas Brasileiros; UEL: Laboratório de Zoonoses e Saúde Pública do Departamento de Medicina Veterinária Preventiva da Universidade Estadual de Londrina; *samples from captivity / unknown exact locality

Number in the map	Species	Sample code	Latitude	Longitude	Sample Type	Source
**1**	*Cebus albifrons*	IPB994	-1.05	-62.89	Tissue	[5]
**2**	*Cebus albifrons*	RETA10	-3.138056	-55.509722	Tissue	M.G.M.L.; unpublished
**3**	*Cebus albifrons*	RETA9	-3.138056	-55.509722	Tissue	[5]
**4**	*Cebus albifrons*	FR85	-6.570031	-64.369217	Tissue	[13]
**5**	*Cebus albifrons*	LB1272	-4.935556	-68.17333333	Tissue	[57]
**6**	*Cebus albifrons*	LB1285	-4.935556	-68.17333333	Tissue	[57]
**7**	*Cebus albifrons*	LB1293	-4.935556	-68.17333333	Tissue	[57]
**8**	*Cebus albifrons*	LB1297	-4.935556	-68.17333333	Tissue	[5]
**9**	*Cebus albifrons*	LB736	-2.47	-64.83	Tissue	[5]
**10**	*Cebus albifrons*	LB772	-2.590944	-64.88627778	Tissue	[13]
**11**	*Cebus albifrons*	LB858	-4.662722	-56.552722	Tissue	[5]
**12**	*Cebus kaapori*	CPB414	*	*	Blood	CPB/ICMBio
**13**	*Cebus kaapori*	CPB443	*	*	Blood	CPB/ICMBio
**14**	*Cebus olivaceus*	AP116	-0.582778	-52.332778	Tissue	[57]
**15**	*Cebus olivaceus*	AP117	-0.582778	-52.332778	Tissue	[13]
**16**	*Cebus olivaceus*	AP178	-3.22	-52.03	Tissue	[5]
**17**	*Cebus olivaceus*	AP241	-3.85	-52.76	Tissue	[13]
**18**	*Cebus olivaceus*	AP242	-3.85	-52.76	Tissue	[57]
**19**	*Cebus olivaceus*	CPB479	*	*	Blood	CPB/ICMBio
**20**	*Saimiri*	SG6118	*	*	Tissue	UT Austin
**21**	*Saimiri*	SP3654	*	*	Tissue	UT Austin
**22**	*Saimiri*	SP4308	*	*	Tissue	UT Austin
**23**	*Sapajus apella*	AP110	-0.307777778	-52.45138889	Tissue	[5]
**24**	*Sapajus apella*	AP133	-0.582777778	-52.33277778	Tissue	[13]
**25**	*Sapajus apella*	AP146	3.220277778	-52.02777778	Tissue	[13]
**26**	*Sapajus apella*	AP162	3.220277778	-52.02777778	Tissue	M.G.M.L.; unpublished
**27**	*Sapajus apella*	AP225	3.853888889	-52.75777778	Tissue	M.G.M.L.; unpublished
**28**	*Sapajus apella*	AP226	3.853888889	-52.75777778	Tissue	M.G.M.L.; unpublished
**29**	*Sapajus apella*	CA2403	-8.89	-63.24	Tissue	[13]
**30**	*Sapajus apella*	CN138	-0.962769444	-55.52224444	Tissue	[5]
**31**	*Sapajus apella*	CN150	-0.165488889	-55.1864	Tissue	[5]
**32**	*Sapajus apella*	CN153	-0.165488889	-55.1864	Tissue	[5]
**33**	*Sapajus apella*	CN217	1.285419444	-58.6959	Tissue	[13]
**34**	*Sapajus apella*	CN235	0.828619444	-53.9312	Tissue	M.G.M.L.; unpublished
**35**	*Sapajus apella*	CN236	-0.83	-53.93	Tissue	M.G.M.L.; unpublished
**36**	*Sapajus apella*	CN249	-0.943969444	-53.2363	Tissue	M.G.M.L.; unpublished

**37**	*Sapajus apella*	CN250	-0.943969444	-53.2363	Tissue	[5]
**38**	*Sapajus apella*	CN292	0.630280556	-55.7285	Tissue	[5]
**39**	*Sapajus apella*	CN293	0.630280556	-55.7285	Tissue	M.G.M.L.; unpublished
**40**	*Sapajus apella*	CTG19	-2.060694444	-58.38461111	Tissue	M.G.M.L.; unpublished
**41**	*Sapajus apella*	CTG30	-1.88	-58.23	Tissue	M.G.M.L.; unpublished
**42**	*Sapajus apella*	FES08	-7.613129	-60.792217	Tissue	[5]
**43**	*Sapajus apella*	JIR4590	-12.45298333	-62.92267222	Tissue	[13]
**44**	*Sapajus apella*	LB807	-2.483333333	-55.96666667	Tissue	M.G.M.L.; unpublished
**45**	*Sapajus apella*	LB808	-2.6	-56.18333333	Tissue	[13]
**46**	*Sapajus apella*	LB859	-4.662722222	-56.55272222	Tissue	M.G.M.L.; unpublished
**47**	*Sapajus apella*	LB860	-4.662722222	-56.55272222	Tissue	M.G.M.L.; unpublished
**48**	*Sapajus apella*	MCB42	-3.36	-51.74	Tissue	[13]
**49**	*Sapajus apella*	PARNA68	-3.884916667	-56.77793333	Tissue	[13]
**50**	*Sapajus apella*	RBG16	-12.56244444	-63.4415	Tissue	[13]
**51**	*Sapajus apella*	RETA01	-3.180077778	-55.80346944	Tissue	[13]
**52**	*Sapajus apella*	RETA06	-3.1175	-55.51583333	Tissue	M.G.M.L.; unpublished
**53**	*Sapajus apella*	RETA2	-2.801111111	-55.57527778	Tissue	M.G.M.L.; unpublished
**54**	*Sapajus apella*	RVR19	-10.00358333	-56.04788889	Tissue	[5]
**55**	*Sapajus apella*	RVR20	-10.00169444	-56.04227778	Tissue	[5]
**56**	*Sapajus apella*	RVR21	-10.00169444	-56.04227778	Tissue	[13]
**57**	*Sapajus apella*	RVR66	-9.60425	-56.016	Tissue	[5]
**58**	*Sapajus apella*	RVR69	-9.603944444	-56.01497222	Tissue	[5]
**59**	*Sapajus apella*	UNIR307	-8.801109167	-63.94994278	Tissue	[13]
**60**	*Sapajus apella*	UNIR324	-9.102089722	-62.88418611	Tissue	[13]
**61**	*Sapajus apella*	UNIR349	-12.00090528	-60.68609278	Tissue	M.G.M.L.; unpublished
**62**	*Sapajus apella*	UNIR351	-11.95273917	-60.68598889	Tissue	[5]
**63**	*Sapajus apella*	UNIR359	-12.033825	-60.66866222	Tissue	[13]
**64**	*Sapajus apella*	UNIR395	-8.781854167	-63.7023625	Tissue	M.G.M.L.; unpublished
**65**	*Sapajus apella*	UNIR412	-8.670666667	-62.36875139	Tissue	[13]
**66**	*Sapajus apella*	UNIR429	-8.191056667	-64.01619694	Tissue	[13]
**67**	*Sapajus apella*	UNIR569	-12.50042111	-63.53052944	Tissue	[5]
**68**	*Sapajus cay*	MSF	-16.05580833	-57.71719167	Tissue	[13]
**69**	*Sapajus cay*	W70	-15.564685	-55.67617	Blood	UEL
**70**	*Sapajus cay*	W72	-15.564685	-55.67617	Blood	UEL
**71**	*Sapajus flavius*	CPB1	-6.57015	-35.131517	Blood	CPB/ICMBio
**72**	*Sapajus flavius*	CPB196	-6.57015	-35.131517	Blood	CPB/ICMBio
**73**	*Sapajus flavius*	CPB2	-6.57015	-35.131517	Blood	CPB/ICMBio
**74**	*Sapajus flavius*	CPB429	-7.012178	-34.956933	Blood	CPB/ICMBio
**75**	*Sapajus flavius*	CPB462	-6.57015	-35.131517	Blood	CPB/ICMBio
**76**	*Sapajus flavius*	CPB463	-7.012178	-34.956933	Blood	CPB/ICMBio
**77**	*Sapajus flavius*	CPB502	-7.012178	-34.956933	Blood	CPB/ICMBio
**78**	*Sapajus flavius*	CPB517	-7.012178	-34.956933	Blood	CPB/ICMBio
**79**	*Sapajus* cf. *flavius*[*1]	CPB527	-6.38901	-35.9421	Blood	CPB/ICMBio

**80**	*Sapajus* cf. *flavius* [*1]	CPB536	-9.530297	-37.858073	Blood	CPB/ICMBio
**81**	*Sapajus flavius*	CPB559	-7.012178	-34.956933	Blood	CPB/ICMBio
**82**	*Sapajus flavius*	CPB570	-7.01510877	-35.09102391	Blood	CPB/ICMBio
**83**	*Sapajus flavius*	CPB580	-7.012178	-34.956933	Blood	CPB/ICMBio
**84**	*Sapajus flavius*	CPB589	-7.476653	-34.984681	Blood	CPB/ICMBio
**85**	*Sapajus flavius*	CPB593	-7.476653	-34.984681	Blood	CPB/ICMBio
**86**	*Sapajus flavius*	CPB598	-7.476653	-34.984681	Blood	CPB/ICMBio
**87**	*Sapajus flavius*	CPB601	-7.476653	-34.984681	Blood	CPB/ICMBio
**88**	*Sapajus flavius*	CPB608	-7.476653	-34.984681	Blood	CPB/ICMBio
**89**	*Sapajus flavius*	CPB613	-7.015639	-35.088806	Blood	CPB/ICMBio
**90**	*Sapajus flavius*	CPB615	-7.015639	-35.088806	Blood	CPB/ICMBio
**91**	*Sapajus flavius*	CPB616	-6.609469	-35.135155	Blood	CPB/ICMBio
**92**	*Sapajus flavius*	CPB617	-6.609469	-35.135155	Blood	CPB/ICMBio
**93**	*Sapajus flavius*	CPB618	-6.609469	-35.135155	Blood	CPB/ICMBio
**94**	*Sapajus libidinosus*	CPB112	-9.010022	-42.691312	Blood	CPB/ICMBio
**95**	*Sapajus libidinosus*	CPB237	-8.736013	-42.636224	Blood	CPB/ICMBio
**96**	*Sapajus libidinosus*	CPB238	-8.736013	-42.636224	Blood	CPB/ICMBio
**97**	*Sapajus libidinosus*	CPB239	-8.736013	-42.636224	Blood	CPB/ICMBio
**98**	*Sapajus libidinosus*	CPB240	-8.736013	-42.636224	Blood	CPB/ICMBio
**99**	*Sapajus libidinosus*	CPB241	-8.736013	-42.636224	Blood	CPB/ICMBio
**100**	*Sapajus libidinosus*	CPB379	-6.600234	-38.249935	Tissue	CPB/ICMBio
**101**	*Sapajus libidinosus*	CPB382	-6.600234	-38.249935	Tissue	CPB/ICMBio
**102**	*Sapajus libidinosus*	CPB447	-6.942634	-38.324782	Tissue	CPB/ICMBio
**103**	*Sapajus libidinosus*	CPB456	-6.765285	-38.23697	Tissue	CPB/ICMBio
**104**	*Sapajus libidinosus*	CPB457	-6.886193	-38.560818	Tissue	CPB/ICMBio
**105**	*Sapajus libidinosus*	CPB458	-6.886193	-38.560818	Tissue	CPB/ICMBio
**106**	*Sapajus libidinosus*	CPB619	-7.949199	-38.282713	Tissue	CPB/ICMBio
**107**	*Sapajus libidinosus*	CPB620	-7.949199	-38.282713	Blood	CPB/ICMBio
**108**	*Sapajus libidinosus*	CPB621	-7.949199	-38.282713	Blood	CPB/ICMBio
**109**	*Sapajus libidinosus*	DT01	-2.7665	-41.80761111	Tissue	[5]
**110**	*Sapajus libidinosus*	DT03	-2.804027778	-41.86638889	Tissue	[5]
**111**	*Sapajus libidinosus*	DT05	-2.846388889	-41.83055556	Tissue	[5]
**112**	*Sapajus libidinosus*	DT06	-2.846388889	-41.83055556	Tissue	M.G.M.L.; unpublished
**113**	*Sapajus libidinosus*	DT8	-7.929077778	-44.19598611	Tissue	[13]
**114**	*Sapajus libidinosus*	LIB01	-5.09	-42.43	Tissue	[5]
**115**	*Sapajus libidinosus*	MN36336	-14.13657222	-48.38323889	Tissue	[5]
**116**	*Sapajus libidinosus*	MN37431	-14.14	-48.17	Tissue	[13]
**117**	*Sapajus libidinosus*	UFG1	-16.60365556	-49.26155	Tissue	[5]
**118**	*Sapajus libidinosus*	UFG2	-16.60365556	-49.26155	Tissue	[13]
**119**	*Sapajus macrocephalus*	AAM3	-3.887694444	-64.25105556	Tissue	[5]
**120**	*Sapajus macrocephalus*	CTG118	-0.613388889	-64.92069444	Tissue	[13]
**121**	*Sapajus macrocephalus*	CTG160	-4.987138889	-62.95977778	Tissue	[13]
**122**	*Sapajus macrocephalus*	CTG173	-5.697	-63.241	Tissue	M.G.M.L.; unpublished
**123**	*Sapajus macrocephalus*	CTG183	-5.694	-63.238	Tissue	[13]
**124**	*Sapajus macrocephalus*	CTG5673	-2.449527778	-65.36355556	Blood	M.G.M.L.; unpublished
**125**	*Sapajus macrocephalus*	CTG5675	-2.449527778	-65.36355556	Blood	M.G.M.L.; unpublished
**126**	*Sapajus macrocephalus*	CTG713	-1.84	-69.03	Tissue	M.G.M.L.; unpublished
**127**	*Sapajus macrocephalus*	CTG715	-1.84	-69.03	Tissue	[13]

**128**	*Sapajus macrocephalus*	FR115	-3.37	-60.48472222	Tissue	[13]
**129**	*Sapajus macrocephalus*	JPB80	-0.478194444	-64.41125	Tissue	[13]
**130**	*Sapajus macrocephalus*	LB1253	-5.209166667	-69.31583333	Tissue	M.G.M.L.; unpublished
**131**	*Sapajus macrocephalus*	LB1265	-5.209166667	-69.31583333	Tissue	[5]
**132**	*Sapajus macrocephalus*	LB1295	-4.935555556	-68.17333333	Tissue	M.G.M.L.; unpublished
**133**	*Sapajus macrocephalus*	LB1298	-4.935555556	-68.17333333	Tissue	[13]
**134**	*Sapajus macrocephalus*	LB138	-4.395869444	-70.13845278	Tissue	[13]
**135**	*Sapajus macrocephalus*	LB139	-4.395869444	-70.13845278	Tissue	M.G.M.L.; unpublished
**136**	*Sapajus macrocephalus*	LB712	-2.469722222	-64.82583333	Tissue	[13]
**137**	*Sapajus macrocephalus*	LB740	-2.590944444	-64.88627778	Tissue	M.G.M.L.; unpublished
**138**	*Sapajus macrocephalus*	LB743	-2.590944444	-64.88627778	Tissue	M.G.M.L.; unpublished
**139**	*Sapajus macrocephalus*	LB751	-2.590944444	-64.88627778	Tissue	[13]
**140**	*Sapajus macrocephalus*	LB767	-2.590944444	-64.88627778	Tissue	M.G.M.L.; unpublished
**141**	*Sapajus macrocephalus*	LB768	-2.590944444	-64.88627778	Tissue	M.G.M.L.; unpublished
**142**	*Sapajus macrocephalus*	LB779	-2.449527778	-65.36355556	Tissue	[5]
**143**	*Sapajus macrocephalus*	LB780	-2.449527778	-65.36355556	Tissue	[13]
**144**	*Sapajus macrocephalus*	SGC	-0.233333333	-66.85	Tissue	[13]
**145**	*Sapajus nigritus*	W112	-22.833333	-53.332778	Blood	UEL
**146**	*Sapajus nigritus*	W25	-22.845833	-53.304444	Blood	UEL
**147**	*Sapajus nigritus*	W59	-22.833333	-53.332778	Blood	UEL
**148**	*Sapajus robustus*	CP2110	*	*	Blood	[13]
**149**	*Sapajus robustus*	CPB165	*	*	Blood	CPB/ICMBio
**150**	*Sapajus robustus*	CPB167	*	*	Blood	CPB/ICMBio
**151**	*Sapajus robustus*	CPB169	*	*	Blood	CPB/ICMBio
**152**	*Sapajus* cf. *flavius* *1	CPB530	-9.392467	-38.201539	Blood	CPB/ICMBio
**153**	*Sapajus* cf. *flavius* *1	CPB531	-9.392467	-38.201539	Blood	CPB/ICMBio
**154**	*Sapajus* cf. *flavius* *1	CPB532	-9.392467	-38.201539	Blood	CPB/ICMBio
**155**	*Sapajus* cf. *flavius* *1	CPB533	-9.392467	-38.201539	Blood	CPB/ICMBio
**156**	*Sapajus* cf. *flavius* *1	CPB534	-9.392467	-38.201539	Blood	CPB/ICMBio
**157**	*Sapajus* cf. *flavius* *1	CPB538	-9.657455	-37.660893	Blood	CPB/ICMBio
**158**	*Sapajus* cf. *flavius* *1	CPB542	-9	-37.91	Blood	CPB/ICMBio
**159**	*Sapajus* cf. *flavius* *1	CPB543	-9	-37.91	Blood	CPB/ICMBio
**160**	*Sapajus* cf. *flavius* *1	CPB544	-9	-37.91	Blood	CPB/ICMBio
**161**	*Sapajus* cf. *flavius* *1	CPB545	-9	-37.91	Blood	CPB/ICMBio
**162**	*Sapajus xanthosternos*	CP1624	-14.79	-39.05	Blood	[13]
**163**	*Sapajus xanthosternos*	CP2006	-14.79	-39.05	Blood	[13]
**164**	*Sapajus xanthosternos*	CPB102	-10.489513	-36.442764	Blood	CPB/ICMBio
**165**	*Sapajus xanthosternos*	CPB103	-10.489513	-36.442764	Blood	CPB/ICMBio
**166**	*Sapajus xanthosternos*	CPB104	-10.489513	-36.442764	Blood	CPB/ICMBio
**167**	*Sapajus xanthosternos*	CPB105	-10.489513	-36.442764	Blood	CPB/ICMBio
**168**	*Sapajus xanthosternos*	CPB107	-10.489513	-36.442764	Blood	CPB/ICMBio
**169**	*Sapajus xanthosternos*	CPB108	-10.489513	-36.442764	Blood	CPB/ICMBio
**170**	*Sapajus xanthosternos*	CPB164	*	*	Blood	CPB/ICMBio
**171**	*Sapajus xanthosternos*	CPB174	*	*	Blood	CPB/ICMBio

*[1]samples from populations of capuchins occupying areas of the Atlantic Forest-Caatinga transition whose taxonomic assignment as *Sapajus flavius* is uncertain.
