# Supplementary material for: A New Assessment of Robust Capuchin Monkey (Sapajus) Evolutionary History Using Genome-Wide SNP Marker Data and a Bayesian Approach to Species Delimitation
Source: Genes (Basel). 2023 Apr 25;14(5):970. doi: 10.3390/genes14050970 (PMC10218464; doi:10.3390/genes14050970)
Supplement: Supplementary file 1 [file genes-14-00970-s001.zip › Table_S3_TotalLociPerSample_ByMissingData.pdf]

Table S3 – Total number of loci recovered for each sample that are present in the final data matrix under varying levels of missing data.

		Total number of loci recovered for each sample by % of missing data						
**Sample code**	**Species**	**≈20% (150 minsp[1])**	**≈25% (130 minsp[1])**	**≈30% (100 minsp[1])**	**≈40% (50 minsp[1])**	**≈50% (30 minsp[1])**	**≈60% (10 minsp[1])**	**≈70% (4 minsp[1])**
FR85	*C. albifrons*	16115	24473	28923	32839	34610	39286	40167
IPB994	*C. albifrons*	13188	18490	21092	23256	24131	26953	27280
LB1272	*C. albifrons*	16362	26474	33099	39178	42225	48973	50377
LB1285	*C. albifrons*	16689	28150	37502	50513	59576	72811	76963
LB1293	*C. albifrons*	16668	27694	35683	44645	50236	59166	61412
LB1297	*C. albifrons*	16627	27282	34546	42764	47219	55000	56962
LB736	*C. albifrons*	16639	27906	36938	48718	55894	66530	69762
LB772	*C. albifrons*	16628	27738	36289	45570	52205	63327	66190
LB858	*C. albifrons*	16492	26881	33635	38652	40387	45804	46755
RETA10	*C. albifrons*	15764	23584	27466	30879	32318	36649	37425
RETA9	*C. albifrons*	8866	11599	13067	14868	15903	18326	19124
CPB414	*C. kaapori*	7848	11008	12365	13410	13881	15649	15921
CPB443	*C. kaapori*	16592	27688	36280	46497	53791	64978	68042
AP116	*C. olivaceus*	16620	27586	35730	44381	49144	57839	60156
AP117	*C. olivaceus*	16195	25622	31699	38230	41604	48101	49709
AP178	*C. olivaceus*	16356	26391	32757	37582	39343	44658	45724
AP241	*C. olivaceus*	15924	24259	28643	32119	33452	37591	38335
AP242	*C. olivaceus*	16592	27562	35566	43549	48078	56725	59032
CPB479	*C. olivaceus*	13231	19279	23219	28457	32234	38927	42649
AP110	*S. apella*	16109	27209	33125	37191	38248	38772	38848
AP133	*S. apella*	16529	28760	36169	41579	43428	44520	44647
AP146	*S. apella*	16083	26301	31352	35086	36687	37638	37781
AP162	*S. apella*	16749	30013	38883	45923	48827	50932	51208
AP225	*S. apella*	14090	23533	28734	32007	33100	33973	34127
AP226	*S. apella*	9926	14122	15636	16422	16639	16824	16850
CA2403	*S. apella*	16225	28677	36848	43434	46376	49168	49733
CN138	*S. apella*	16828	30676	41148	51643	55500	57778	58088
CN150	*S. apella*	16497	28710	35590	39285	40131	40666	40732
CN153	*S. apella*	16474	29290	38078	45848	48735	50526	50895
CN217	*S. apella*	11320	18057	21209	22911	23332	23698	23796
CN235	*S. apella*	16080	27752	35127	41725	45127	48033	48508
CN236	*S. apella*	7268	11005	12807	14096	14638	15025	15116
CN249	*S. apella*	16844	30557	40517	50439	56098	61045	61837
CN250	*S. apella*	16788	30305	39113	44526	45857	46719	46835
CN292	*S. apella*	16870	31116	42934	55882	61522	65866	66787
CN293	*S. apella*	16860	30974	41701	50416	53333	55440	55670
CTG19	*S. apella*	16840	30732	40993	49557	54150	58651	59236
CTG30	*S. apella*	16828	30575	40347	49276	53350	56338	56888
FES08	*S. apella*	16858	31126	43379	60519	76757	98495	102571
JIR4590	*S. apella*	16869	31126	43241	58467	66077	72244	73087
LB807	*S. apella*	16552	29593	38828	47432	52767	57950	58936
LB808	*S. apella*	16869	31115	43000	57740	65985	73316	74495
LB859	*S. apella*	16498	28916	36667	41857	43502	44655	44807
LB860	*S. apella*	16869	31002	42002	52962	59258	64905	65669
MCB42	*S. apella*	16678	29610	37752	43047	44413	45197	45298
PARNA68	*S. apella*	8641	13292	15321	16527	17006	17397	17504

Table S3 continued

RBG16	*S. apella*	13377	19625	22245	24167	24714	25054	25104
RETA01	*S. apella*	16871	31125	43074	58217	67410	74811	75849
RETA06	*S. apella*	13137	21690	26159	28675	29346	29913	30057
RETA2	*S. apella*	16312	28287	35196	38828	39499	39903	39946
RVR19	*S. apella*	16851	30928	41936	51232	53432	54799	55068
RVR20	*S. apella*	16842	30693	40519	47433	49725	51585	51855
RVR21	*S. apella*	16816	30309	38814	44273	47029	49501	49868
RVR66	*S. apella*	16863	31026	42016	51527	55773	59183	59528
RVR69	*S. apella*	16716	30002	39473	47604	52056	56440	57180
UNIR307	*S. apella*	11186	18033	21519	23813	24713	25431	25606
UNIR324	*S. apella*	16787	30290	39910	50439	56958	62639	63862
UNIR349	*S. apella*	16872	31063	42467	53239	56344	58189	58579
UNIR351	*S. apella*	16855	30923	41783	51342	53751	55115	55475
UNIR359	*S. apella*	16850	30839	41193	49413	52423	54632	54896
UNIR395	*S. apella*	11341	16652	18639	19552	19746	19845	19865
UNIR412	*S. apella*	16805	30699	41273	52608	61076	70354	72300
UNIR429	*S. apella*	7027	9373	10171	10714	10836	10906	10924
UNIR569	*S. apella*	16867	31138	43321	59838	70479	79949	81891
MSF	*S. cay*	16666	29375	36629	40695	41571	42083	42162
W70	*S. cay*	16859	31125	43329	60943	72991	83265	85253
W72	*S. cay*	16868	31142	43393	61437	76734	92726	95996
CPB1	*S. flavius*	7713	12666	15572	17897	18959	19779	19924
CPB196	*S. flavius*	16647	28895	35530	39542	40572	41186	41258
CPB2	*S. flavius*	16867	30999	42547	55718	61571	66537	67239
CPB429	*S. flavius*	16860	31034	42355	54502	63241	72109	73545
CPB462	*S. flavius*	16865	31094	42680	55213	65612	76433	77939
CPB463	*S. flavius*	16805	30639	41252	51788	58403	64745	65447
CPB502	*S. flavius*	16851	30972	42315	54728	62585	69725	70569
CPB517	*S. flavius*	16796	30567	40763	49397	52701	55342	55600
CPB527	*S. flavius*	16852	31026	42604	54692	60996	66517	67126
CPB536	*S. flavius*	16793	30238	39695	48203	53853	59692	60865
CPB559	*S. flavius*	16639	28979	35864	40700	42593	43930	44108
CPB570	*S. flavius*	16874	31152	43387	60839	74317	86513	88428
CPB580	*S. flavius*	16871	31136	43368	60500	71850	83091	85464
CPB589	*S. flavius*	16856	31044	42638	55079	63672	72452	73813
CPB593	*S. flavius*	16868	31124	43280	60144	71349	81659	83838
CPB598	*S. flavius*	16666	28974	35891	40253	41622	42538	42682
CPB601	*S. flavius*	15392	24218	28475	31462	32467	33049	33111
CPB608	*S. flavius*	16868	31110	43156	58352	72491	90350	93380
CPB613	*S. flavius*	16865	31132	43274	58924	72718	90883	94154
CPB615	*S. flavius*	16861	31100	42985	57383	68344	79961	81727
CPB616	*S. flavius*	11249	16377	18326	19297	19569	19789	19822
CPB617	*S. flavius*	16851	30714	40499	49093	53198	56254	56716
CPB618	*S. flavius*	16858	30766	40796	49320	53316	56016	56302
CPB112	*S. libidinosus*	12598	20001	24456	29687	32933	36474	38854
CPB237	*S. libidinosus*	16865	31112	43161	58521	72267	88918	91878
CPB238	*S. libidinosus*	16860	31103	43147	58606	71249	85762	88352
CPB239	*S. libidinosus*	16861	31019	42574	55397	63831	72584	73842
CPB240	*S. libidinosus*	16843	30949	41992	52623	58950	64651	65297
CPB241	*S. libidinosus*	16852	30988	41991	52328	57607	62197	62820
CPB379	*S. libidinosus*	16861	31055	42633	55080	61848	67379	68009
CPB382	*S. libidinosus*	16832	30685	40505	48041	51137	53793	54102
CPB447	*S. libidinosus*	16857	31076	42974	57909	70318	84443	87249

Table S3 continued

CPB456	*S. libidinosus*	16836	30625	40658	48820	52301	54939	55239
CPB457	*S. libidinosus*	16317	28380	36072	42663	45993	48581	48920
CPB458	*S. libidinosus*	16666	29011	35992	40835	42796	44264	44456
CPB619	*S. libidinosus*	14491	23366	27737	30903	32342	33436	33701
CPB620	*S. libidinosus*	16871	31134	43270	59209	73863	91056	93927
CPB621	*S. libidinosus*	16860	31033	42143	53094	63294	75178	77011
DT01	*S. libidinosus*	16726	30113	39657	48164	53415	58863	59895
DT03	*S. libidinosus*	16854	31074	43241	60564	74133	88990	92032
DT05	*S. libidinosus*	16672	29870	39138	47480	52622	58072	58941
DT06	*S. libidinosus*	16855	31067	43192	61035	80445	108967	116444
DT8	*S. libidinosus*	16851	30971	42163	53883	60853	67296	68175
LIB01	*S. libidinosus*	13426	20947	23991	25291	25578	25776	25813
MN36336	*S. libidinosus*	8282	13102	15595	17321	18089	18727	18919
MN37431	*S. libidinosus*	16846	30808	41162	49622	53327	56356	56754
UFG1	*S. libidinosus*	16864	31070	42885	58005	66452	73286	74277
UFG2	*S. libidinosus*	15949	25943	31024	34714	35819	36457	36563
AAM3	*S. macrocephalus*	4864	7528	8821	9846	10231	10435	10481
CTG118	*S. macrocephalus*	16737	30272	40018	48775	53562	58226	59101
CTG160	*S. macrocephalus*	16862	31007	42122	51042	52818	53832	54011
CTG173	*S. macrocephalus*	16872	31035	42013	51615	56577	61683	62566
CTG183	*S. macrocephalus*	16162	26702	32681	38678	42073	45192	46544
CTG5673	*S. macrocephalus*	16856	30898	41992	54352	63634	73555	75845
CTG5675	*S. macrocephalus*	16669	29841	38839	46264	50531	54949	55801
CTG713	*S. macrocephalus*	16866	31124	43204	58647	67133	73714	74785
CTG715	*S. macrocephalus*	16867	31105	42874	57578	65731	72448	74896
FR115	*S. macrocephalus*	14574	22228	25750	28167	28807	29145	29201
JPB80	*S. macrocephalus*	11058	15543	17052	17871	18157	18369	18399
LB1253	*S. macrocephalus*	16833	30565	40528	50001	55151	59977	60737
LB1265	*S. macrocephalus*	14446	22387	25601	27182	27572	27808	27846
LB1295	*S. macrocephalus*	16874	31145	43128	57495	67987	78864	80394
LB1298	*S. macrocephalus*	16310	27277	33507	39120	41524	43208	43552
LB138	*S. macrocephalus*	16771	29619	37723	45131	48577	51120	51603
LB139	*S. macrocephalus*	16783	30293	39686	46719	49277	51528	51841
LB712	*S. macrocephalus*	16808	30145	39438	49242	55675	61022	62157
LB740	*S. macrocephalus*	16872	31149	43244	59719	73346	89158	92191
LB743	*S. macrocephalus*	16870	31102	42953	56678	62526	67025	67695
LB751	*S. macrocephalus*	12334	20175	24319	26932	27967	28888	29078
LB767	*S. macrocephalus*	16870	31139	43284	61481	83082	122324	141689
LB768	*S. macrocephalus*	16873	31120	42937	55829	62090	66359	66819
LB779	*S. macrocephalus*	16865	31109	43140	59316	71092	83848	86501
LB780	*S. macrocephalus*	15552	25069	29523	32247	33220	33827	33899
SGC	*S. macrocephalus*	16090	26444	31756	35235	36233	36771	36835
W112	*S. nigritus*	16713	30732	42570	60080	74680	90002	94320
W25	*S. nigritus*	8175	12437	15068	18516	21185	23936	25939
W59	*S. nigritus*	4971	6865	7625	8262	8669	9062	9249
CP2110	*S. robustus*	16753	30369	40200	49700	54419	58463	59534
CPB165	*S. robustus*	16781	30530	40400	48569	52441	55703	56543
CPB167	*S. robustus*	16639	29743	38378	44404	46389	48241	48872
CPB169	*S. robustus*	16775	30723	41497	52095	57690	62535	63585
CP1624	*S. xanthosternos*	16781	30807	42340	56784	69696	85748	89134
CP2006	*S. xanthosternos*	16768	30821	42562	57594	72451	93764	98416
CPB102	*S. xanthosternos*	13325	20502	24213	27896	29799	31584	32459
CPB103	*S. xanthosternos*	16681	30337	40287	48413	52258	55665	56827

Table S3 continued

CPB104	*S. xanthosternos*	16708	30462	41143	53394	62108	70868	73149
CPB105	*S. xanthosternos*	16556	29162	37291	44784	49140	52877	54054
CPB107	*S. xanthosternos*	16688	30424	40936	51788	59372	66658	68584
CPB108	*S. xanthosternos*	16702	30577	41925	54871	66116	78550	81633
CPB164	*S. xanthosternos*	6725	10486	12843	15967	18752	22255	25100
CPB174	*S. xanthosternos*	16715	30551	41195	51163	56436	61269	62444
SG6118	*Saimiri*	9018	14751	19079	24443	28981	34591	51694
SP3654	*Saimiri*	9160	15091	19763	25998	31785	38994	56894
SP4308	*Saimiri*	8859	14389	18418	22844	26187	30751	47471
CPB530	*Sapajus sp.*	16863	30878	41442	50965	55234	58285	58665
CPB531	*Sapajus sp.*	16828	30696	41165	50696	55655	60002	60536
CPB532	*Sapajus sp.*	10657	15934	18229	19385	19699	19941	19973
CPB533	*Sapajus sp.*	16841	30850	41565	51906	56955	60790	61200
CPB534	*Sapajus sp.*	16776	30063	38954	45656	48355	50223	50417
CPB538	*Sapajus sp.*	16787	29793	38380	47054	52546	57240	58027
CPB542	*Sapajus sp.*	16703	29639	38013	43590	45371	46626	46747
CPB543	*Sapajus sp.*	16856	30771	40990	49378	52906	55288	55517
CPB544	*Sapajus sp.*	16798	30219	38880	45826	49356	52331	52723
CPB545	*Sapajus sp.*	16875	31131	42949	56055	63107	68600	69243

[1]Minimum number of samples a locus must be present in to be included in the final genotype matrix, represented as a percentage of the total number of samples.
