# Supplementary figures and images for: A New Assessment of Robust Capuchin Monkey (Sapajus) Evolutionary History Using Genome-Wide SNP Marker Data and a Bayesian Approach to Species Delimitation
Source: Genes (Basel). 2023 Apr 25;14(5):970. doi: 10.3390/genes14050970 (PMC10218464; doi:10.3390/genes14050970)

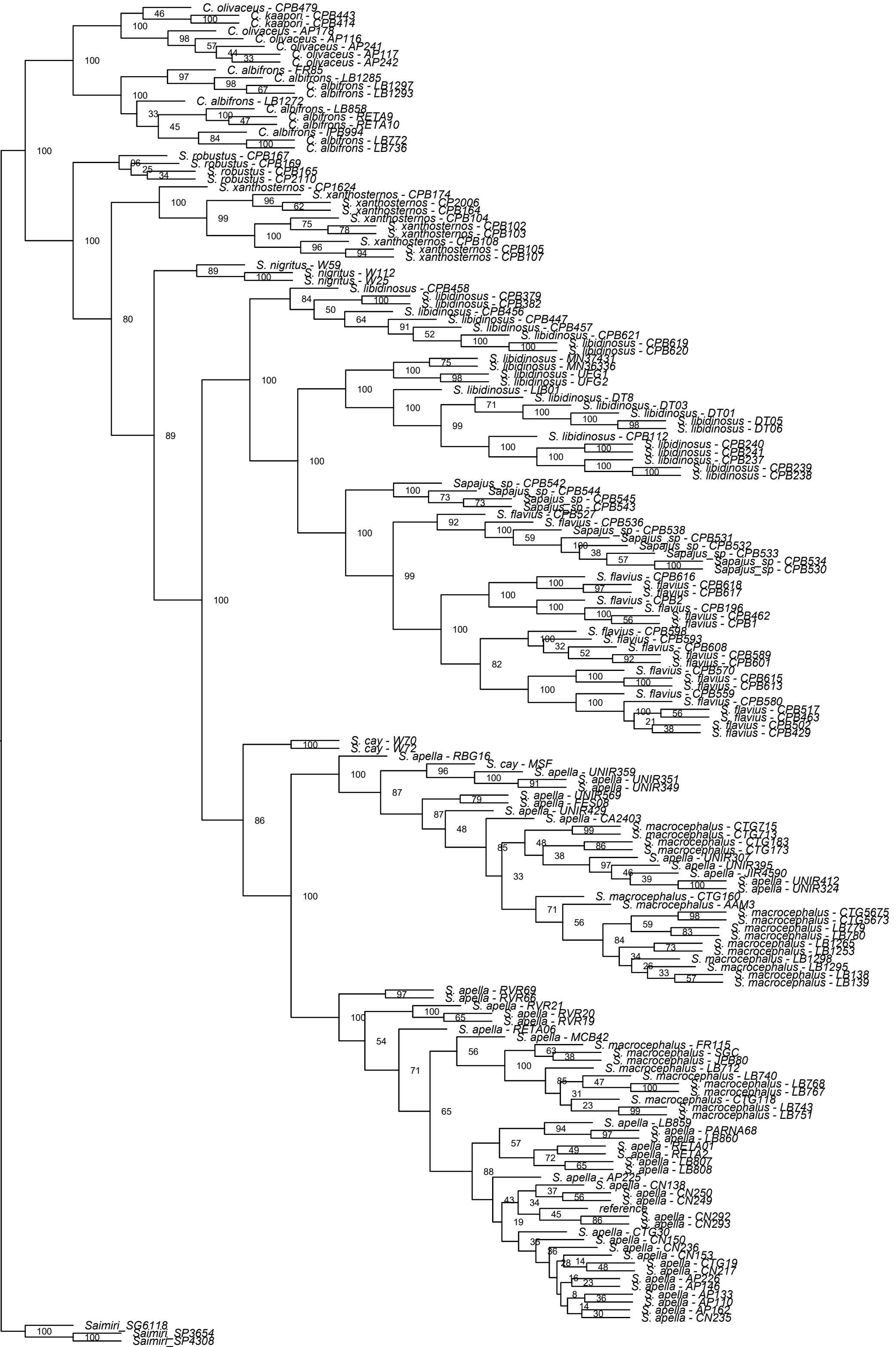

200.0

Supplement: Supplementary file 1 [file genes-14-00970-s001.zip › Figure_S10_Tetrad_100minsp__1000btp.pdf]

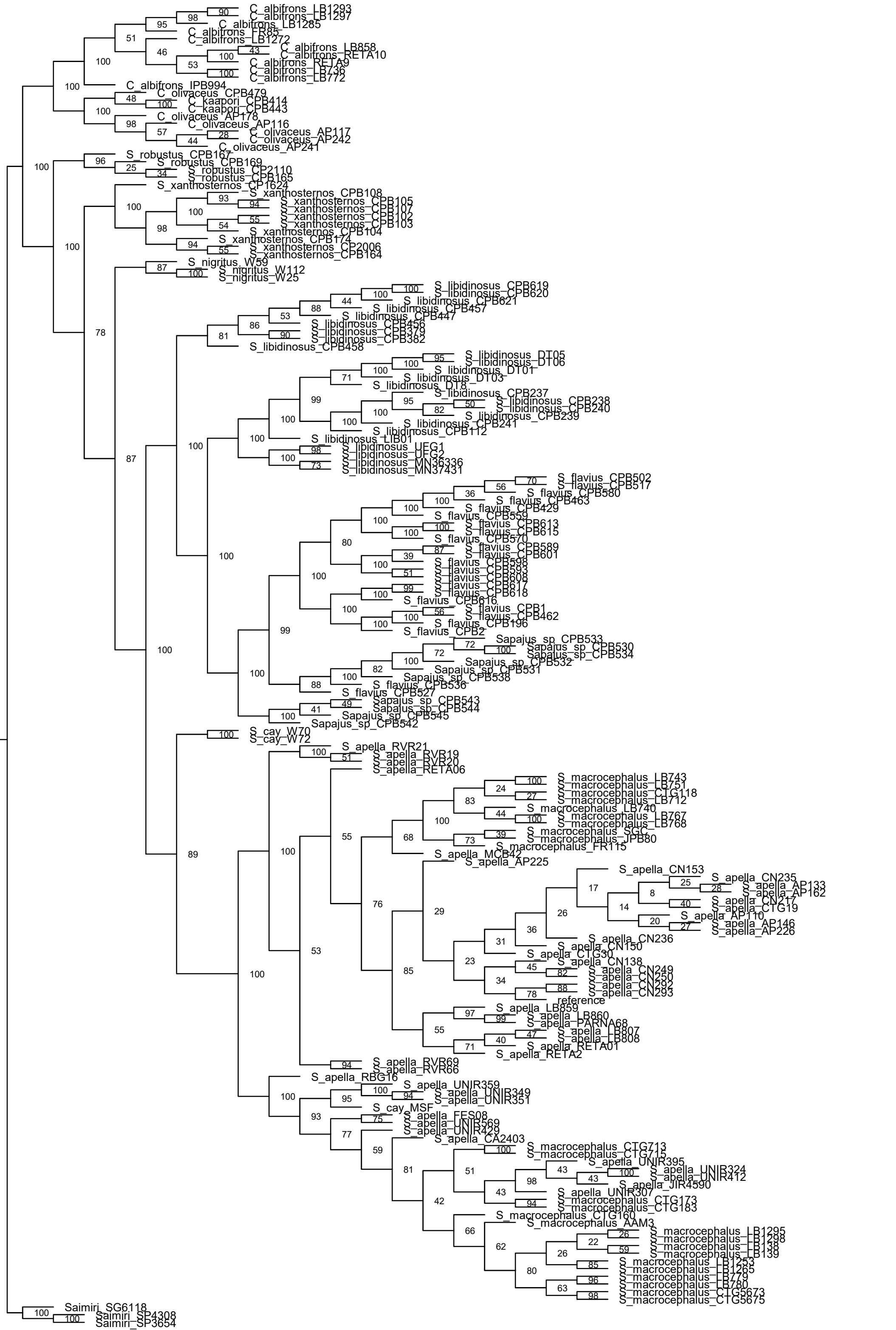

3.0

Supplement: Supplementary file 1 [file genes-14-00970-s001.zip › Figure_S11_Tetrad_130minsp__1000btp.pdf]

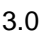

Supplement: Supplementary file 1 [file genes-14-00970-s001.zip › Figure_S12_Tetrad_150minsp_1000btp.pdf]

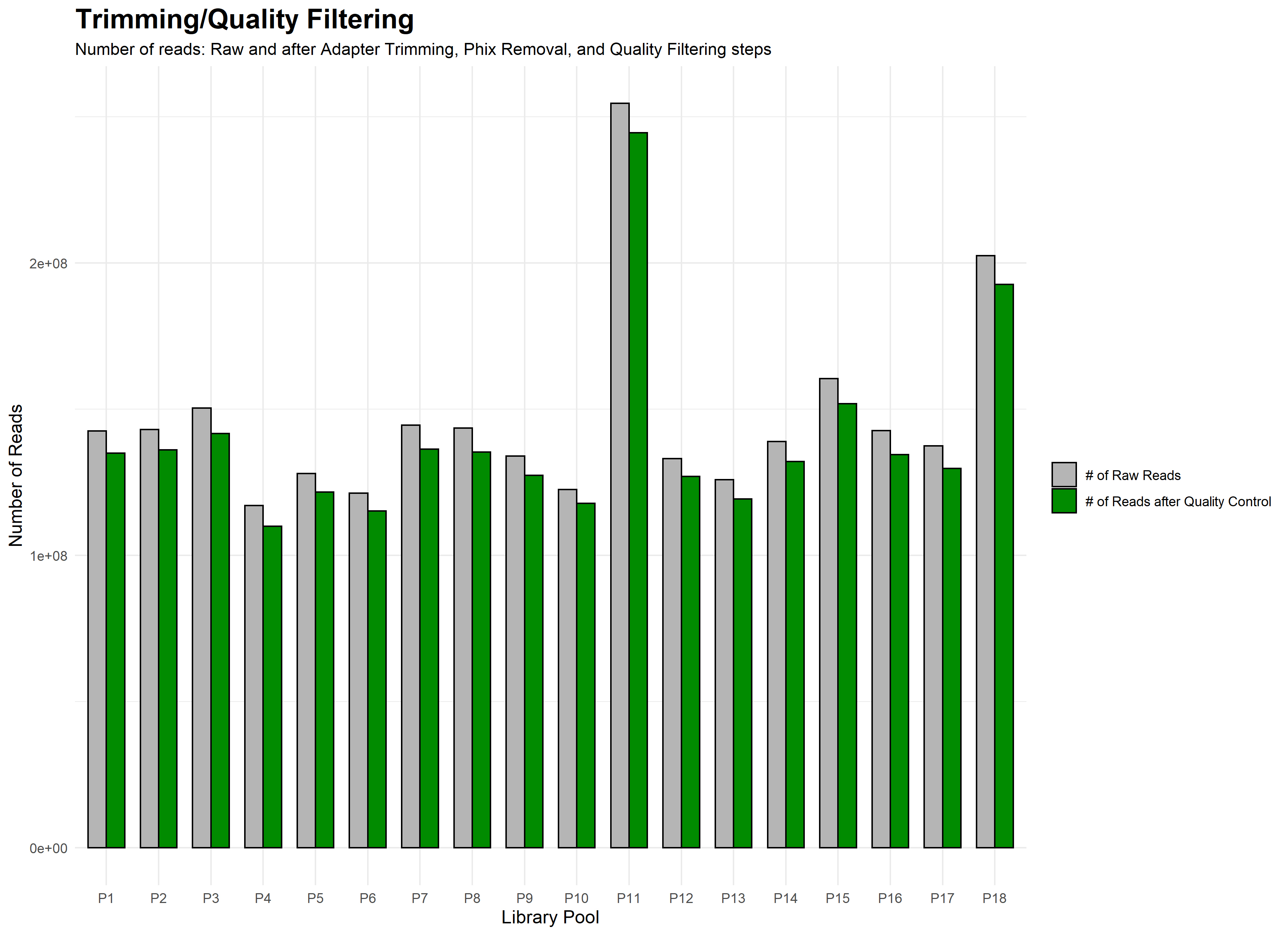

Supplement: Supplementary file 1 [file genes-14-00970-s001.zip › Figure_S1_Raw_FilteredReads5.jpg]

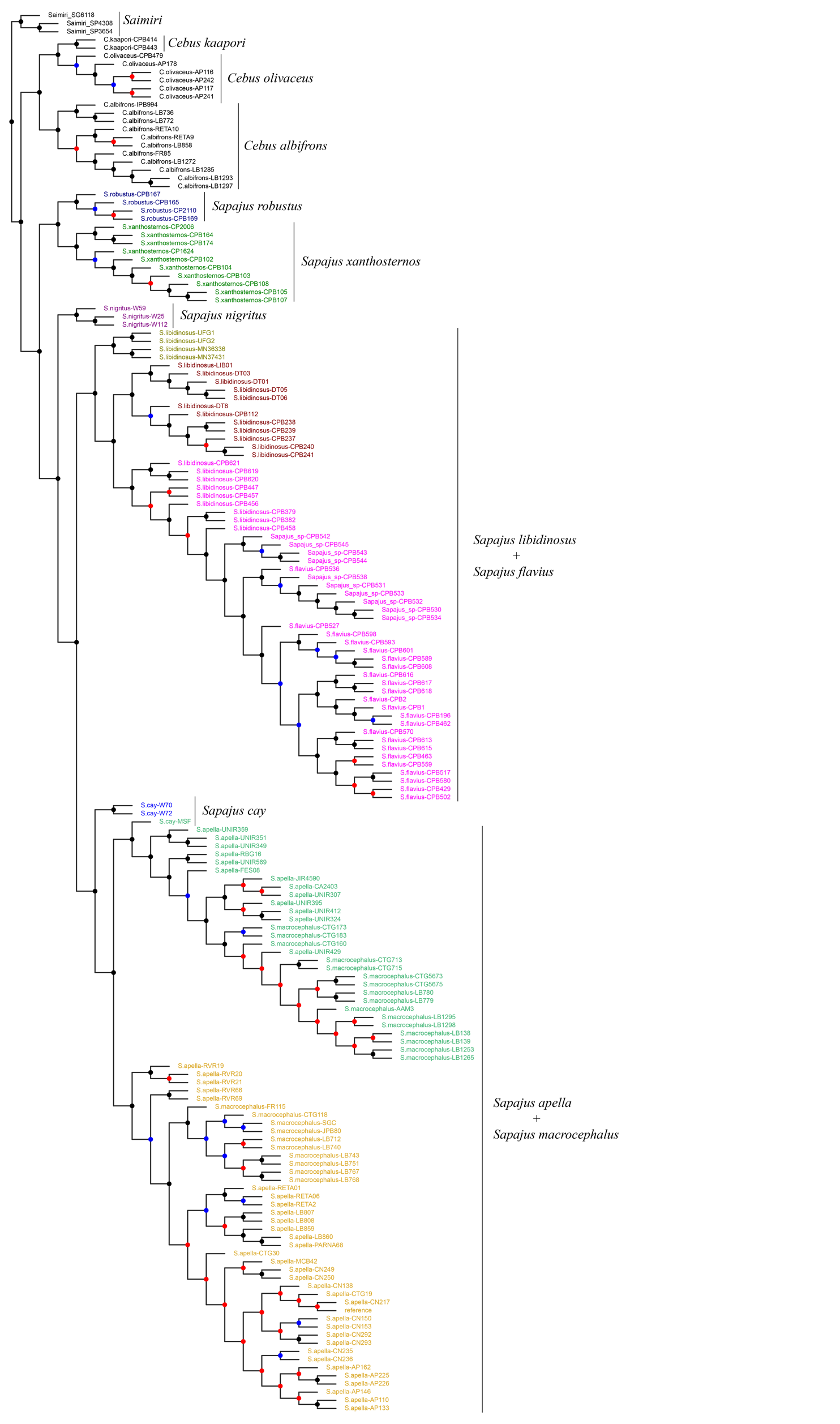

Supplement: Supplementary file 1 [file genes-14-00970-s001.zip › Figure_S2.jpg]

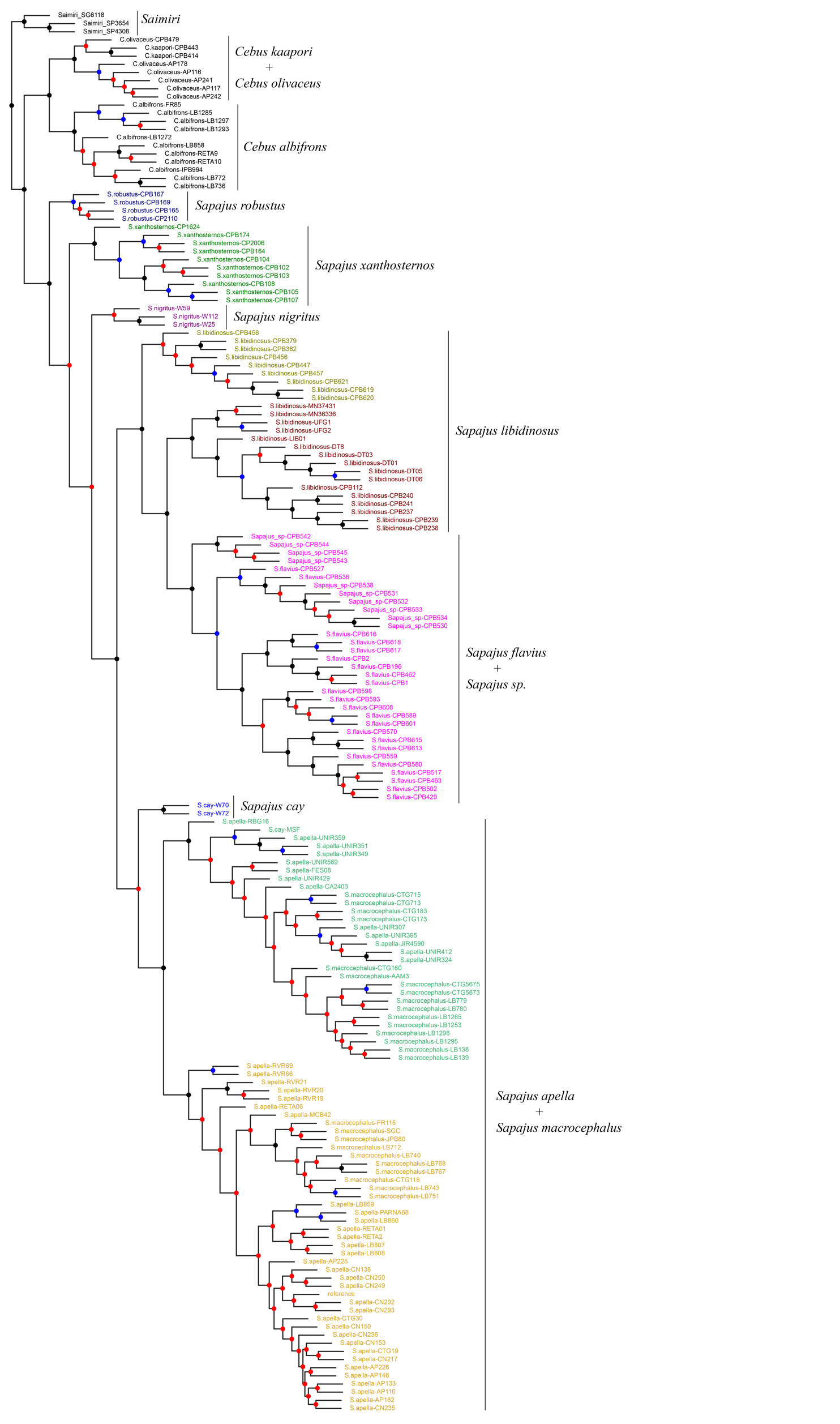

Supplement: Supplementary file 1 [file genes-14-00970-s001.zip › Figure_S3.jpg]

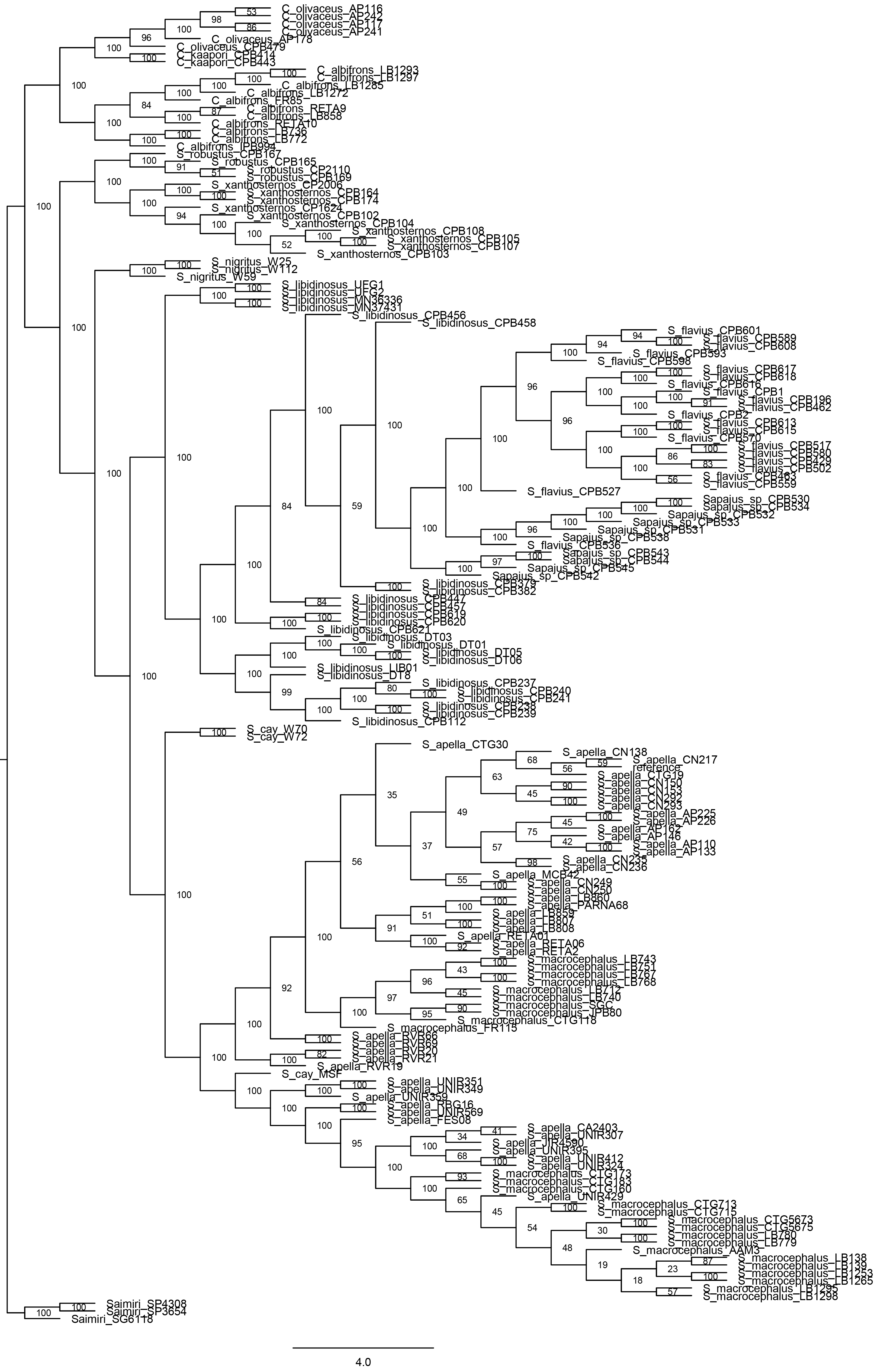

Supplement: Supplementary file 1 [file genes-14-00970-s001.zip › Figure_S4_ML_IQTREE_AllSupportValues.jpg]

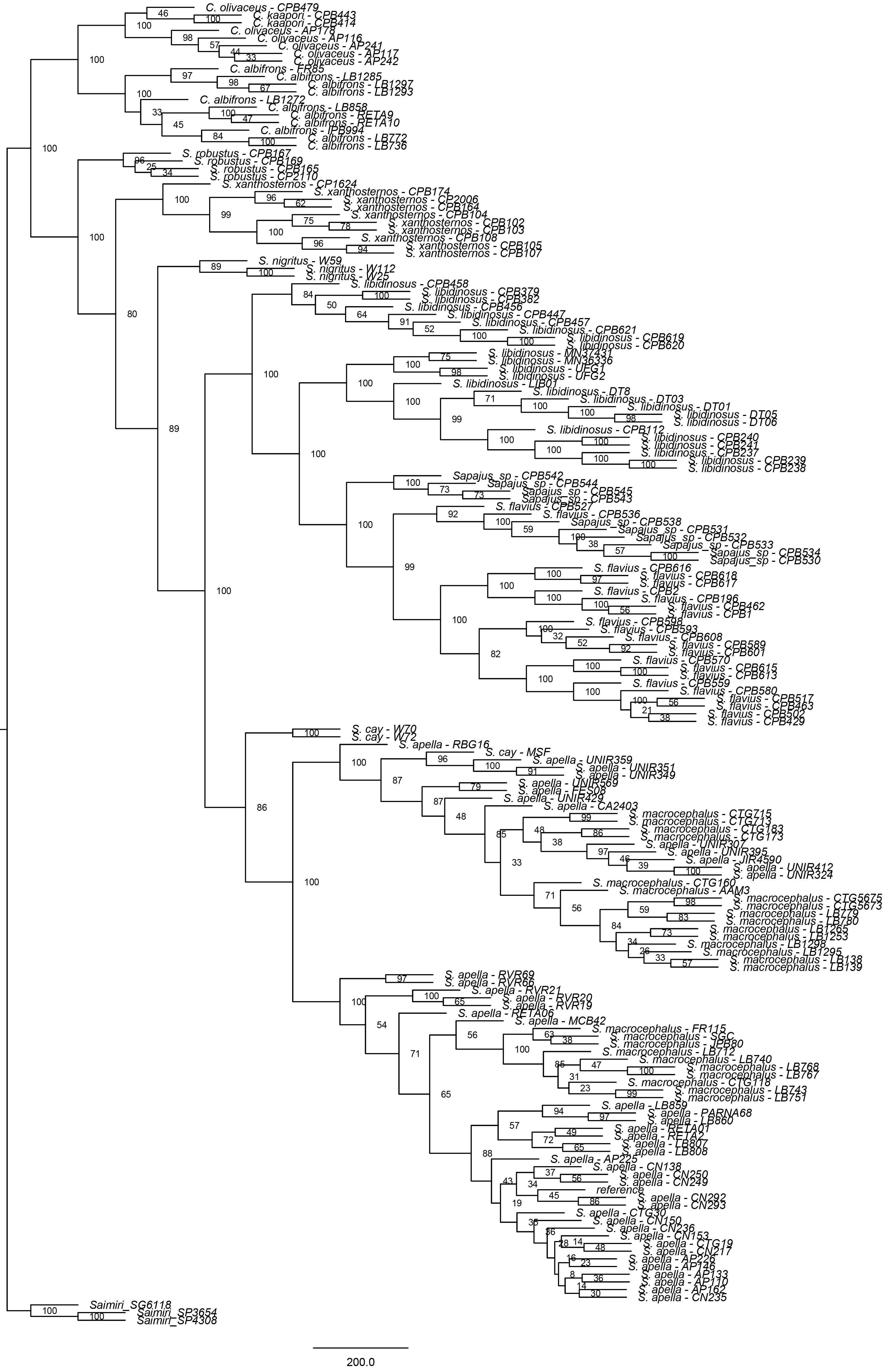

Supplement: Supplementary file 1 [file genes-14-00970-s001.zip › Figure_S5_MSC_Tetrad_AllSupportValues.jpg]

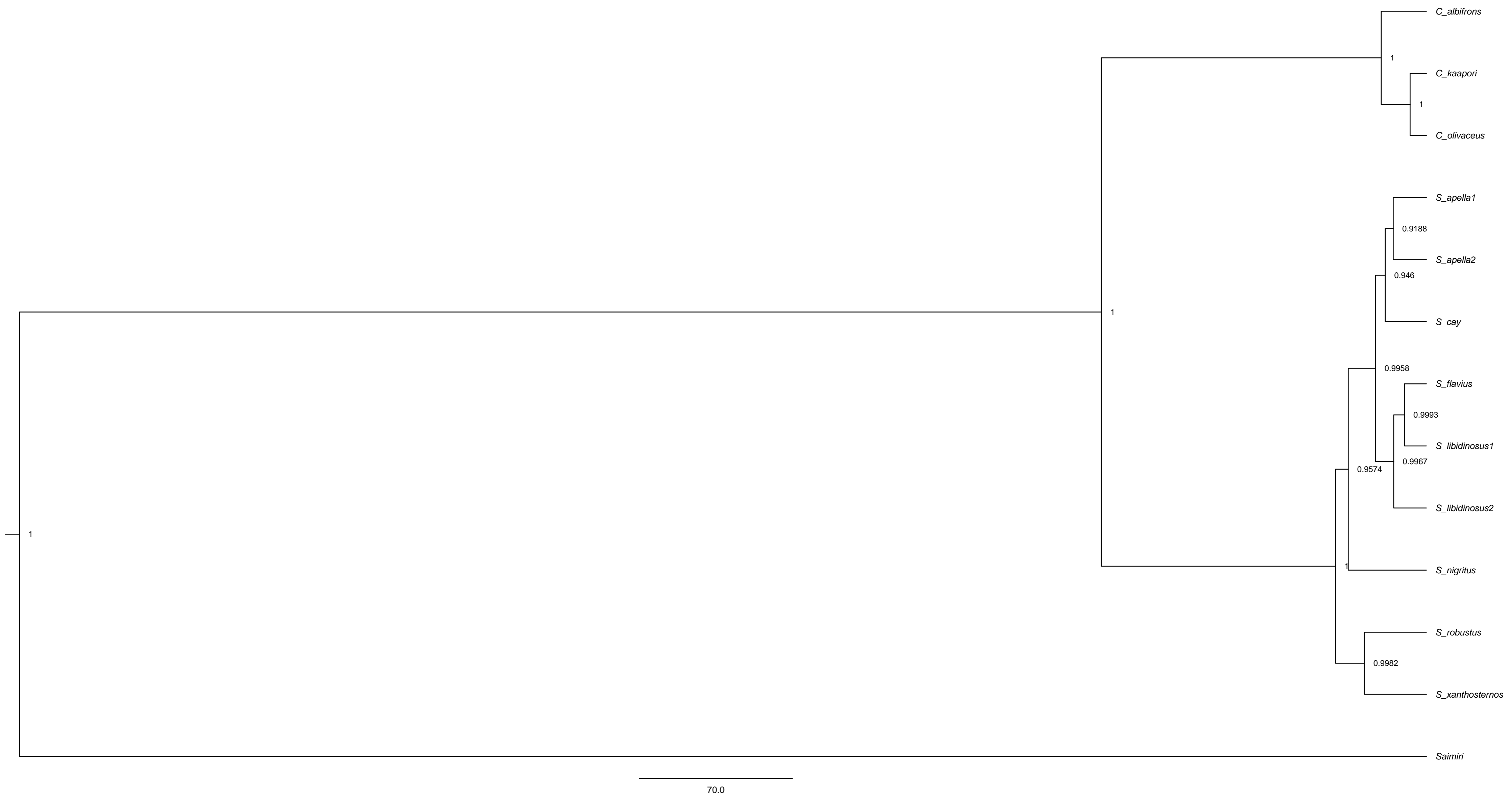

Supplement: Supplementary file 1 [file genes-14-00970-s001.zip › Figure_S6_SBeast_Summary_tree_MeanHeights.pdf]

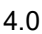

## 4.0

Supplement: Supplementary file 1 [file genes-14-00970-s001.zip › Figure_S7_IQTREE_Phylog_100minsp_cons_1000btp.pdf]

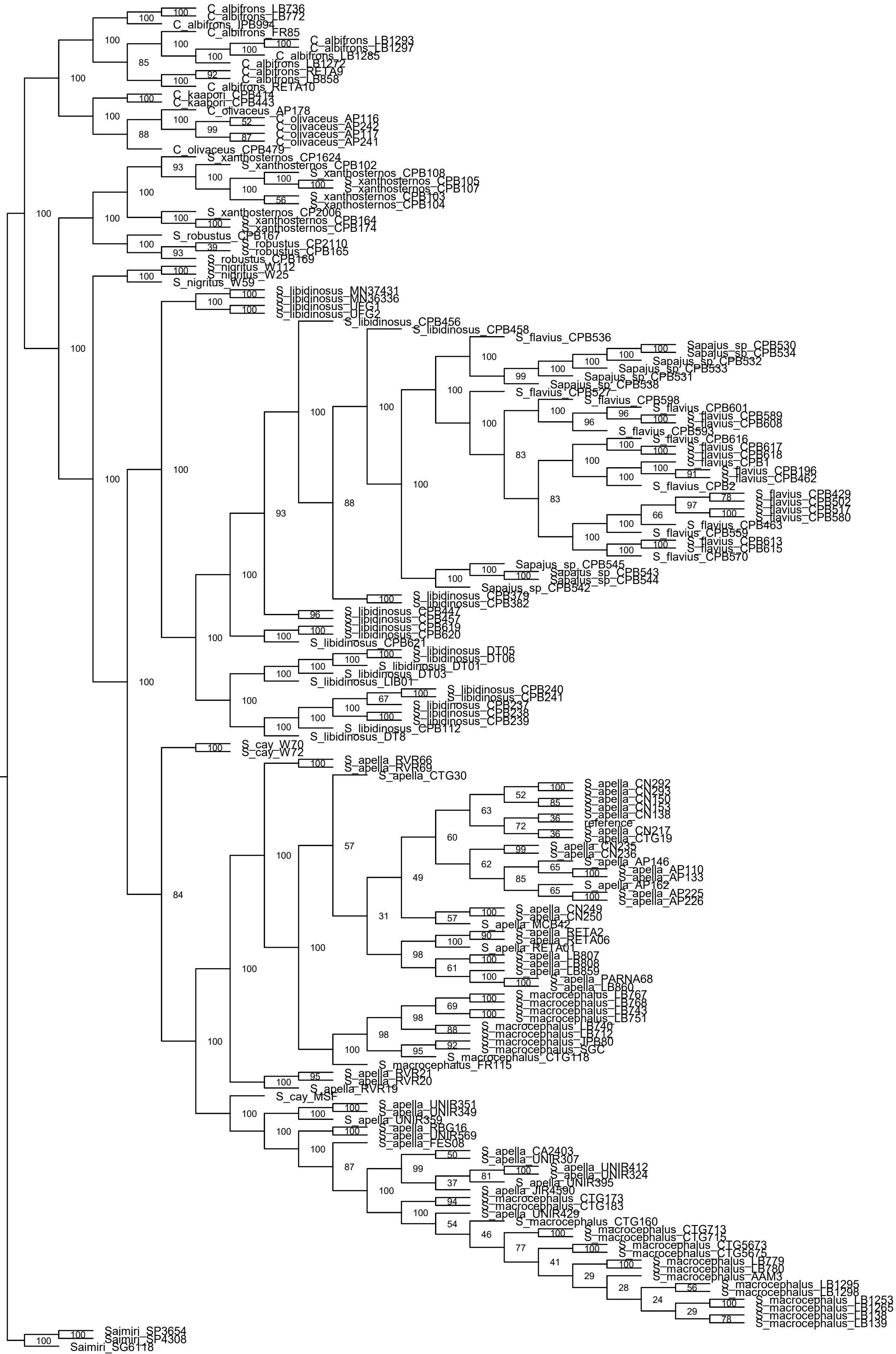

4.0

Supplement: Supplementary file 1 [file genes-14-00970-s001.zip › Figure_S8_IQTREE_Phylog_130minsp_cons_1000btp.pdf]

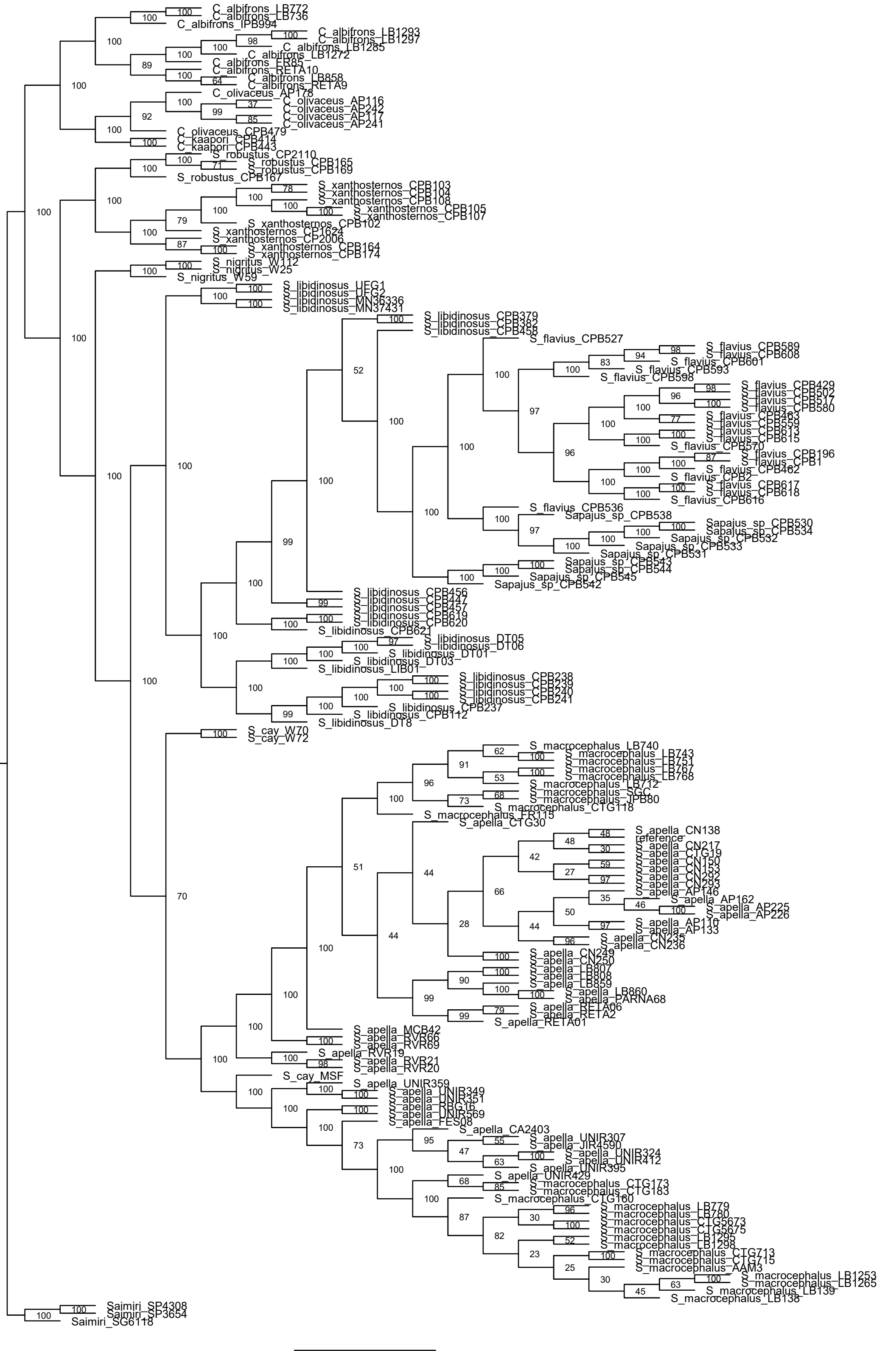

Supplement: Supplementary file 1 [file genes-14-00970-s001.zip › Figure_S9_IQTREE_Phylog_150minsp_cons_1000btp.pdf]
